# Effect of IRAK-M on Airway Inflammation Induced by Cigarette Smoking

**DOI:** 10.1155/2017/6506953

**Published:** 2017-08-29

**Authors:** Haihong Gong, Tao Liu, Wei Chen, Weixun Zhou, Jinming Gao

**Affiliations:** ^1^Department of Respiratory Diseases, Peking Union Medical College Hospital, Chinese Academy of Medical Sciences & Peking Union Medical College, Beijing 100730, China; ^2^Department of Cardiology, Peking Union Medical College Hospital, Chinese Academy of Medical Sciences & Peking Union Medical College, Beijing 100730, China; ^3^Department of Pathology, Peking Union Medical College Hospital, Chinese Academy of Medical Sciences & Peking Union Medical College, Beijing 100730, China

## Abstract

**Background:**

IRAK-M, negatively regulating Toll-like receptor, is shown the dual properties in the varied disease contexts. We studied the effect of IRAK-M deficiency on cigarette smoking- (CS-) induced airway inflammation under acute or subacute conditions in a mouse model.

**Methods:**

A number of cellular and molecular techniques were used to detect the differences between IRAK-M knockout (KO) and wild type (WT) mice exposed to 3-day or 7-week CS.

**Results:**

Airway inflammation was comparable between IRAK-M KO and WT mice under 3-day CS exposure. Upon short-term CS exposure and lipopolysaccharide (LPS) inhalation, IRAK-M KO mice demonstrated worse airway inflammation, significantly higher percentage of Th17 cells and concentrations of proinflammatory cytokines in the lungs, and significantly elevated expression of costimulatory molecules CD40 and CD86 by lung dendritic cells (DCs) or macrophages. Conversely, 7-week CS exposed IRAK-M KO mice demonstrated significantly attenuated airway inflammation, significantly lower concentrations of proinflammatory cytokines in the lungs, significantly increased percentage of Tregs, and lower expression of CD11b and CD86 by lung DCs or macrophages.

**Conclusions:**

IRAK-M plays distinctive effect on CS-induced airway inflammation, and influences Treg/Th17 balance and expression of costimulatory molecules by DCs and macrophages, depending on duration and intensity of stimulus.

## 1. Introduction

Cigarette smoking (CS) is a recognized risk factor for several airway inflammatory diseases, particularly chronic obstructive pulmonary disease (COPD) [1]. CS impairs the physical integrity and immunodefense functions of airway epithelium, leading to increased susceptibility to bacterial infection of the airways [1, 2]. CS is also a profound proinflammatory stimulus that triggers airway inflammation by activating innate and adaptive immune cells, such as dendritic cells (DCs), macrophages, and T cells. T cells are one of the predominant cell types in the pathogenesis of COPD, with CD4^+^ and CD8^+^ T cells being seen in both the airways and parenchyma of COPD patients [3, 4]. COPD-associated Th1 cells, known as Th17 cells, have been found in the lungs of COPD patients. Evidence from animal model showing the imbalance of T helper 17 cells (Th17)/T-regulatory cells (Treg) induced by CS supported the role of Treg and Th17 cells in the immunopathogenesis of CS-induced lung pathology [5].

Toll-like receptors (TLRs), expressed on airway epithelium, recognize lipopolysaccharide (LPS) which activates intracellular molecules, such as myeloid differentiation factor 88 (MyD88) and IL-1 receptor-associated kinases (IRAKs) that lead to overproduction of proinflammatory cytokines [6]. IRAK-M, known as IRAK-3, is one of IRAK family members and functions as a negative regulator of TLR-mediated NF*κ*B activation. Expression of IRAK-M is located in both airway epithelial cells and monocytes/macrophages of the healthy lungs [7, 8].

Previous studies reported the different effects of IL-6 and IL-22 on lung pathologies during inflammatory and repairing processes [9–11]. IRAK-M has also shown dual properties in various disease contexts. Induction of IRAK-M expression in some certain settings may be helpful in attenuating pathologies by limiting overproduction of proinflammatory cytokines and overactivation of innate immune response [6]. Chen et al. have reported that upregulation of IRAK-M in cardiac macrophages alleviates myocardial inflammation and prevents adverse cardiac remodeling in mouse models of myocardial infarction [12]. However, IRAK-M overexpression in some circumstances can increase host susceptibility to infectious- or noninfectious injury. In vitro studies showed that overexpression of airway epithelial IRAK-M inhibited innate immunity of airway epithelium against bacterial infection and increased epithelial infection to human rhinovirus- (HRV-) 16 [13, 14]. IRAK-M knockout (KO) mice exhibit accentuated inflammatory responses after bacterial and viral infections [7, 15, 16]. Interestingly, recent evidence showed that IRAK-M KO mice had attenuated lung fibrosis induced by bleomycin with a significant reduction of proinflammatory IL-13 in the airways [17].

Given these inconsistent effects of IRAK-M in regulating body immune responses to the environmental insults, its role in cigarette smoking- (CS-) induced airway inflammation remains open. In this study, we reported the distinctive effects of IRAK-M on airway inflammation in the various phases of CS exposure and type of stimulus possibly through influencing the surface expression of costimulatory molecules by DCs and macrophages of the lungs by using IRAK-M KO mice and differentiation of naïve T cells.

## 2. Materials and Methods

### 2.1. Mice

Wild-type (WT) mice with C57BL/6 background were purchased at age of 6 weeks from the Experimental Animal Research Center (Beijing, China). Mice deficient in IRAK-M (B6.129S1-Irak3^tmlFlv^/J), originally from Jackson Laboratory (Bar Harbor, ME), were gifted by Dr. Nikolaos G. Frangogiannis and bred on B6 background for 10 backcrosses [7]. All animals were maintained in mouse facility at Peking Union Medical College Hospital (PUMCH). 8- to 10-week-old mice (~20 grams of weight) were used for all experiments. Mice were age-matched in acute CS exposure group and subacute CS exposure groups. The ages of mice were 12–15 weeks at the end of CS exposure.

All experiments were carried out according to international and institutional guidelines for animal care and approved by Peking Union Medical College Hospital Ethics Committee for animal experimentation.

### 2.2. Cigarette Smoke (CS) Exposure and LPS Inhalation

Mice were exposed to CS in a whole-body exposure system according to our previously described [18]. Briefly, mice were placed in a closed plastic box connected to a smoke generator. To establish acute or subacute animal model of airway inflammation induced by CS or LPS [18, 19], the mice were exposed to tobacco smoke of five cigarettes (reference cigarette 3R4F, University of Kentucky, USA) four times a day with 30 min smoke-free intervals between each smoke exposure for 3 days or for 7 weeks. Control mice were exposed to room air (RA). On the fourth day, some mice were challenged with PBS or 100 *μ*g/ml of LPS (*Escherichia coli* serotype 0111:B4, Sigma) by inhalation for 30 mins according to previously described with minor modification [20–22]. LPS has been applied to replicate animal model of COPD [19]. Mice were sacrificed 24 hours after the last challenge with LPS or CS for further analysis.

### 2.3. Airway Resistance Test

Airway resistance (Rn) was determined as previously described for the invasive analysis of lung function using a computer-controlled small animal ventilator, the Flexivent system (Scireq, Montreal, PQ, Canada) [23].

### 2.4. Bronchoalveolar Lavage (BAL)

Mice were euthanized, and BAL was performed as previously described method. Briefly, cells were obtained from BAL fluids, and cytospins (Thermo Electron, Waltham, MA) were prepared to determine the numbers of total inflammatory cells and differential cells using a modified Wright-Giemsa staining. At least 400 cells were counted for one sample [18, 23].

### 2.5. Lung Histology and Semiquantitative Scorings of Airway Inflammation

For histology samples, lungs were perfused with saline and inflated with 4% paraformaldehyde at 25 cm H2O overnight after the last challenge. Hematoxylin & eosin (H&E) staining was performed at the Department of Pathology, Peking Union Medical College Hospital.

An index of pathological changes of airway inflammation in H&E slides was assessed in a blind manner by scoring the inflammatory cell infiltrated around airways and vessels according to the previously published methods [24, 25]. Briefly, a score of 0–3 on each section was used to reflect overall extent of airway inflammation (0: normal; 1: <25% of each section; 2: 25–75%; and 3: >75%).

### 2.6. Flow Cytometry Analysis

To prepare single cell suspensions, lungs were perfused with 20 ml cold PBS through the right ventricle, carefully minced, digested with collagenase type 1A and type IV bovine pancreatic DNase, and passed through a cell strainer. For detection of surface expression of costimualtory molecules, cells were stained with fluorochrome (FITC, PE, PerCP-Cyanine5.5, APC)-conjugated Abs (anti-mouse CD3, CD4, CD8, F480, CD11b, CD11c, CD40, CD80, CD83, and CD86) for 30 mins at 4°C according to previously published [26]. For measurement of expression of intracellular cytokines, cells were incubated with 50 ng/ml of PMA, 500 ng/ml of ionomycin, and GolgiStop (BD Biosciences) for 5 h at 37°C. Then, cells were stained with anti-CD3 and anti-CD8 for 30 min and next stained with mAbs (anti-IL-17A, anti-IL-4, anti-IFN*γ*, and anti-FOXP3) for 1 h. After wash by PBS containing 0.1% sodium azide, cells were subjected on FACSCalibur (BD Biosciences). All fluorochrome-conjugated Abs were purchased from eBioscience or Biolegend, and the corresponding isotype control Abs were added to “isotype samples.”

### 2.7. Quantification of Cytokines

The left lungs were harvested and homogenized in 1 ml PBS containing protease inhibitor cocktail (Sigma, St. Louis, MO) and centrifuged. Supernatants were filtered and kept at −80°C until analysis. Concentrations of cytokines were quantified by multiplex analysis kits as per the manufacturer's instruction (Biolegend, San Diego, CA). The detection limits of IL-1*α*, IL-1*β*, IL-6, IL-10, IL-12p70, IL-17*α*, IL-23, IL-27, IFN*β*, IFN*γ*, MCP-1, TNF*α*, and GM-CSF were as follows: 1.78 pg/ml, 1.55 pg/ml, 1.27 pg/ml, 1.89 pg/ml, 1.68 pg/ml, 1.73 pg/ml, 8.19 pg/ml, 10.57 pg/ml, 10.95 pg/ml, 1.76 pg/ml, 1.8 pg/ml, 2.89 pg/ml, and 10.69 pg/ml, respectively.

### 2.8. RNA Extraction and Quantitative RT-PCR Analysis (qRT-PCR)

Total RNA of the lung was extracted using TRIzol reagent according to the manufacturer's instructions. Then, cDNA was synthesized using a commercial cDNA kit (Takara Corporation, Japan). qRT-PCR was used to measure mRNA expression of RORC, FOXP3, GATA, and T-bet. The primers for qRT-PCR were listed in Table 1.

### 2.9. Statistical Analysis

Data are expressed as the mean ± SEM. Comparisons between multiple groups were carried out using analysis of variance (ANOVA), while comparisons between two groups were performed by Student's *t-*test. All data analyses were adopted by GraphPad PRISM software (version 6.0 for Windows; GraphPad, San Diego, CA, USA). *p* < 0.05 was considered significant.

## 3. Results

### 3.1. Effect of IRAK-M Loss on Airway Inflammation in Mice Challenged with LPS after Acute Exposure to CS

**Figure 1:**
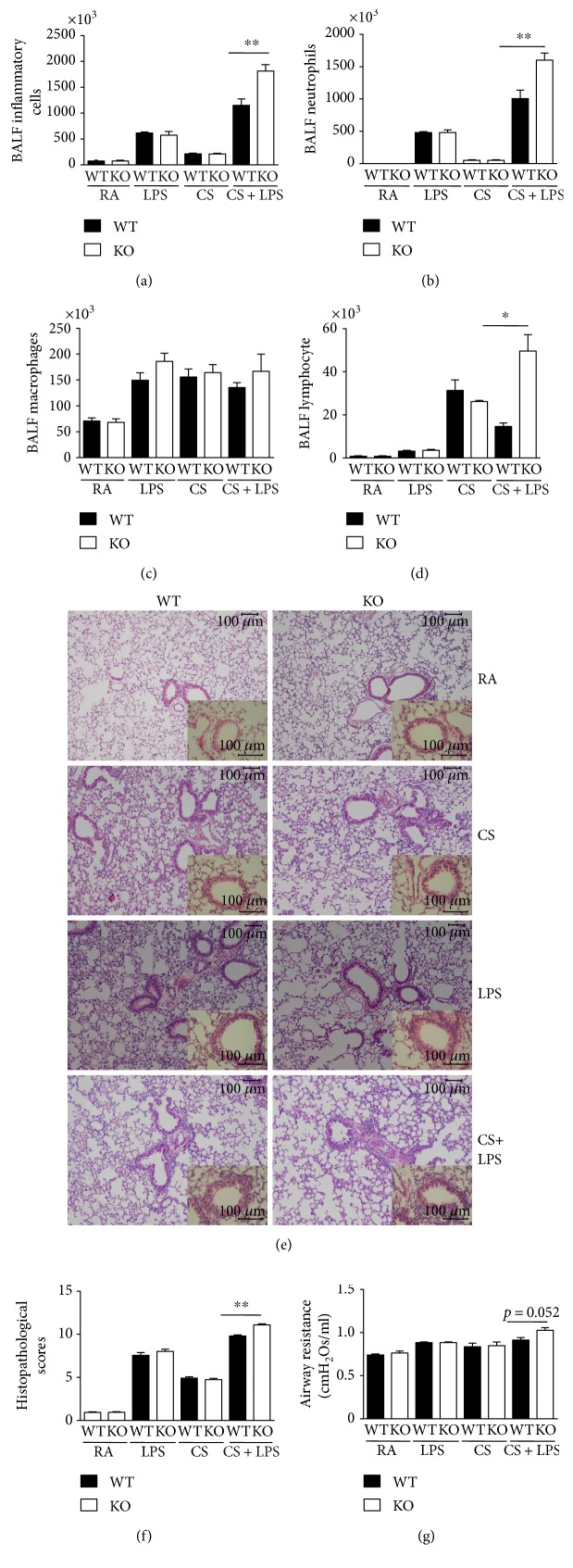
Increased airway inflammation in IRAK-M KO mice after 3-day CS exposure followed by LPS challenge. (a)–(d) The total inflammatory cells and differential populations recovered from BAL fluid. (e) Representative photomicrographs of hematoxylin & eosin-stained lung tissues showing that LPS/short-term CS-insulted IRAK-M^−/−^ mice exhibited the typical pathological characteristics of airway inflammation evidenced by thickened airway epithelium and more inflammatory cells in the peribronchial area and around vessels compared with the similarly treated WT mice. (f) Semiquantative scorings of histopathologic changes. (g) IRAK-M KO mice showed marginally increased airway resistance compared with WT mice after combined exposure to short-term CS and LPS. Results are expressed as means ± SEM, *n* = 5–8 animals per group, ^∗^*p* < 0.05, ^∗∗^*p* < 0.01.

We evaluated the role of IRAK-M in airway inflammation in mice exposed to 3-day CS or inhaled LPS. Comparable airway inflammation (BAL inflammatory cells, aggregation of inflammatory cells around the airways and blood vessels, concentrations of cytokines, and airway resistance) was seen in both IRAK-M^−/−^ and WT mice challenged with either short-term CS or a single dosage of LPS (Figures 1(a), 1(b), 1(c), 1(d), 1(e), 1(f), and 1(g)).

In order to test the differences in airway inflammation between IRAK-M KO mice and their WT counterparts, we next challenged mice with LPS inhalation after 3-day CS exposure [22]. Compared with the WT mice, IRAK-M^−/−^ mice demonstrated more obvious lung inflammation evidenced by more inflammatory cells infiltrated in the airways (BAL total inflammatory cells, neutrophils, and lymphocytes) (Figures 1(a), 1(b), 1(c), and 1(d)) and deposited around peribronchus and vascular (Figure 1(e)). There was a significant increase in inflammation scores in IRAK-M KO mice compared with WT mice (Figure 1(f)). Airway resistance was marginally elevated in IRAK-M^−/−^ mice relative to WT mice (Figure 1(g)).

### 3.2. Effect of IRAK-M Deficiency on T Cell Differentiation in Mice Challenged with LPS plus Short-Term CS Exposure

**Figure 2:**
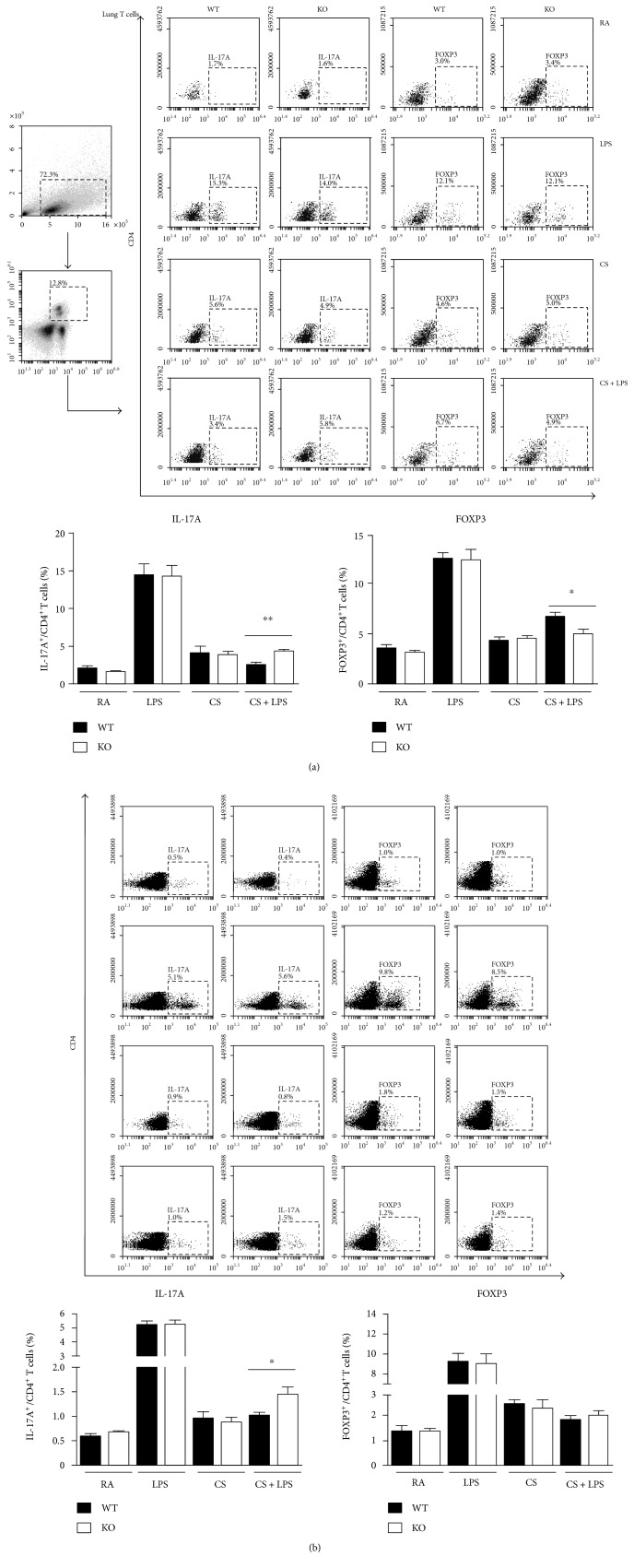
Effect of IRAK-M loss on T subsets after 3-day CS exposure followed by LPS challenge. FACS analysis of Th17 and Treg cells using specific Abs against Th17 and Treg markers in the lung (a) and spleen (b), the percentages of Th17 and Treg were presented as plotted. Results are expressed as means ± SEM, *n* = 7–8 animals per group, ^∗^*p* < 0.05, ^∗∗^*p* < 0.01.

We next examined whether IRAK-M loss influenced Treg/Th17 imbalance in mice challenged with LPS after 3-day CS exposure. The percentage of Tregs (represented by CD3^+^CD4^+^FOXP3^+^T) was significantly lower in lungs from IRAK-M^−/−^ mice than that from WT mice; conversely, the percentage of Th17 (CD3^+^CD4^+^Th17A^+^T) cells was significantly higher in lungs from IRAK-M KO mice than that from WT mice (Figure 2(a)). Under the stimulation with LPS following short CS exposure, we also observed a significant decrease in the percentage of Tregs in the spleen of IRAK-M KO mice compared to WT mice. A mild elevation of the percentage of Th17 in the spleen of IRAK-M KO mice challenged with LPS and acute CS was observed (Figure 2(b)).

### 3.3. Effect of IRAK-M Loss on Cytokine Production in Mice Challenged with LPS after Acute Exposure to CS

**Figure 3:**
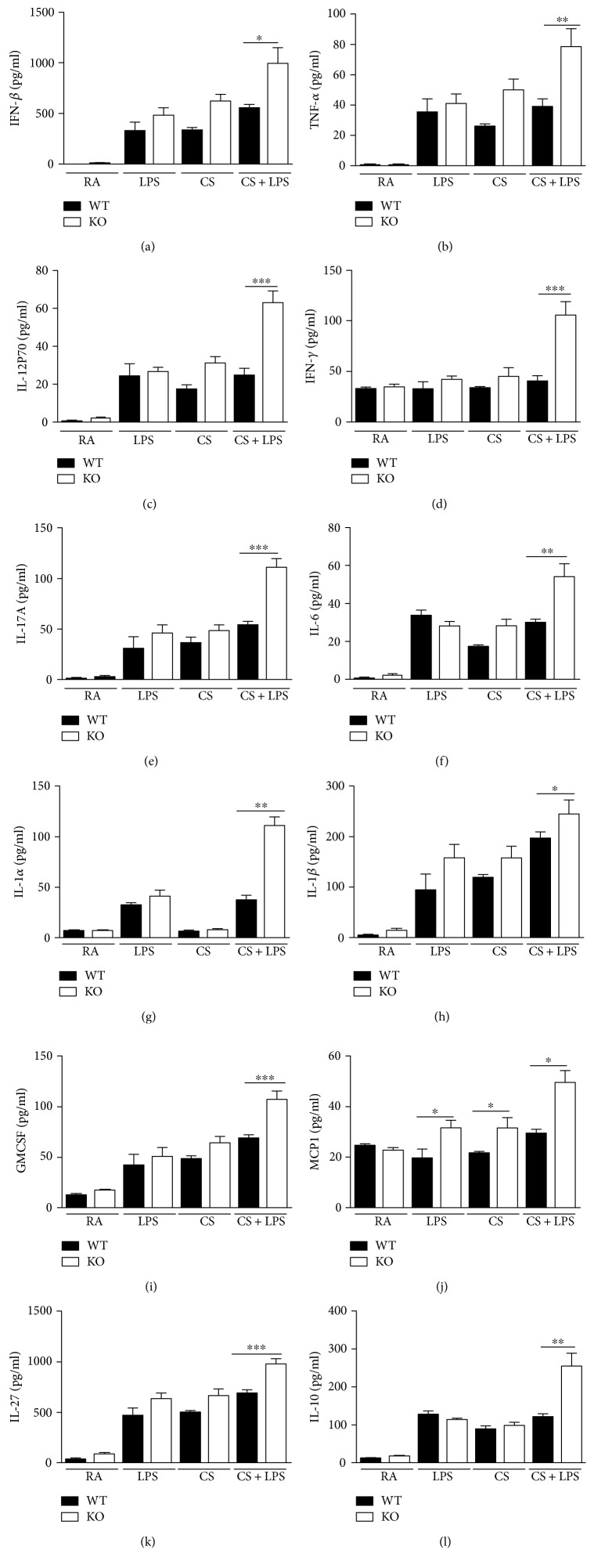
Elevated levels of inflammatory cytokines in lung homogenates in IRAK-M KO mice after 3-day CS exposure followed by LPS challenge. LEGENDplex analysis for levels of cytokines in lung homogenates. (a) Th1-associated cytokines, (b) Th17-related cytokines, (c) proinflammatory cytokines and mediators, and (d) anti-inflammatory cytokines. Results are expressed as means ± SEM, *n* = 7–10 animals per group, ^∗^*p* < 0.05, ^∗∗^*p* < 0.01, ^∗∗∗^*p* < 0.001.

Previous study reported that cells deficient in IRAK-M had elevated secretion of Th1 skewing cytokines by DCs after TLR stimulation [27]. We challenged mice with LPS inhalation after 3-day CS exposure and observed that IRAK-M KO mice showed significantly higher levels of Th1-related cytokines (including TNF*α*, IL-12p70, IFN*β*, and IFN*γ*) in lung homogenates than their WT littermates (Figure 3(a)). Consistently, significantly higher concentrations of Th17-associated cytokines (including IL-17A and IL-6) were seen in lung homogenates from IRAK-M KO mice after smoke-induced exacerbations of LPS infection (Figure 3(b)).

Because IRAK-M is also expressed by airway resident cells [8], we then measured the proinflammatory mediators released by airway epithelium. As shown in Figure 3(c), significantly elevated concentrations of epithelium-derived proinflammatory cytokines and chemokines (including GM-CSF, IL-1*α*, IL-1*β*, and MCP-1) were found in lung homogenates from IRAK-M^−/−^ mice compared with WT mice. Additionally, we also observed the significant elevation of anti-inflammatory cytokines IL-10 and IL-27 in lung homogenates from IRAK-M KO mice, reflecting the nonspecific activation of inflammatory response to LPS and acute exposure to CS (Figure 3(d)).

### 3.4. Effect of IRAK-M Loss on Surface Expression of Costimulatory Molecules by DCs and Macrophages in Mice Challenged with LPS Inhalation following 3-Day CS Exposure

**Figure 4:**
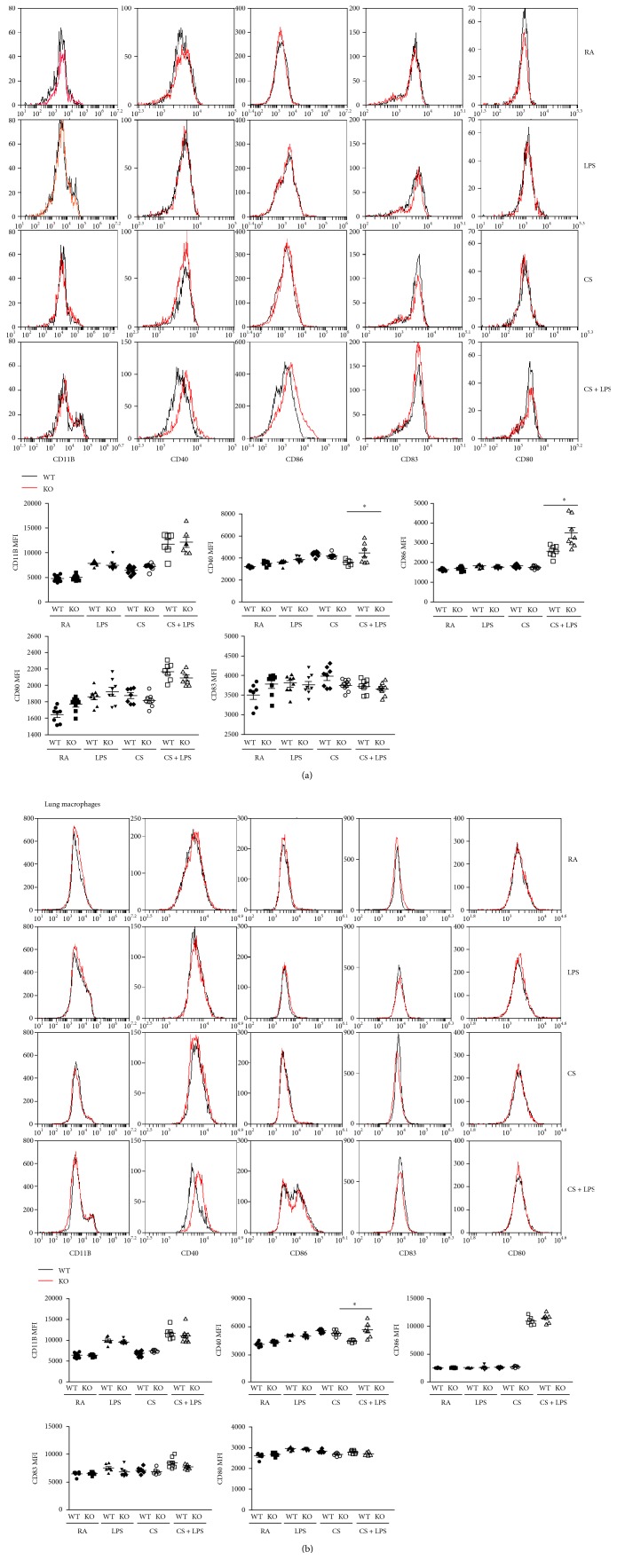
Effect of IRAK-M loss on surface expression of costimulatrory molecules on lung DCs and macrophages after 3-day CS exposure followed by LPS challenge. FACS analysis of surface expression of costimulatory molecules on (a) lung DCs and (b) macrophages using specific Abs against the indicated costimulatory molecule. Mean fluorescence intensity of indicated costimulatory molecules was plotted. Results are expressed as means ± SEM, ^∗^*p* < 0.05.

Dendritic cells (DCs), which function as not only innate lung sentinels but also orchestrators of adaptive immunity, are capable of initiating and maintaining the pathogenesis of COPD [28]. Lung DCs express IRAK-M that has been shown to bind CD80 and further inhibits activation of intracellular NF-*κ*B/AP-1 [29]; we tested the effect of IRAK-M absence on surface expression of costimulatory molecules on lung DCs and macrophages in mice exposed to 3-day CS followed by LPS challenge using FACS analysis. There was significantly increased upregulation of costimulatory molecules CD40 and CD86 on lung DCs from IRAK-M KO mice compared to WT mice (Figure 4(a)).

In addition, significantly increased expression of CD40 by lung macrophages was seen in IRAK-M KO mice following LPS inhalation after 3-day CS insult compared with WT littermates treated under the same exposure condition (Figure 4(b)).

### 3.5. Effect of IRAK-M Ablation on Airway Inflammation in Mice under Subacute Exposure to CS

**Figure 5:**
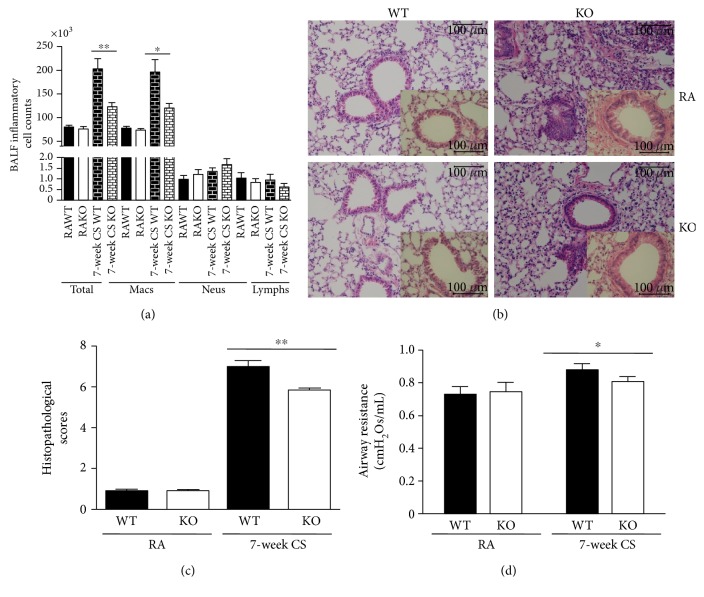
Attenuated airway inflammation and airway resistance, and lower concentration of cytokines in IRAK-M^−/−^ mice after 7-week CS exposure. (a) The total inflammatory cells and differential populations recovered from BAL fluid. (b) Representative photomicrographs of hematoxylin & eosin-stained lung tissues showing that after 7-week CS exposure, IRAK-M KO mice exhibited attenuated pathological characteristics of airway inflammation evidenced by less thickened airway epithelium and less inflammatory cells in the peribronchial area and around vessels compared with the similarly treated WT mice. (c) IRAK-M KO mice showed significantly lower airway resistance compared with WT mice. Results are expressed as means ± SEM, *n* = 5–8 animals per group, ^∗^*p* < 0.05, ^∗∗^*p* < 0.01.

To reflect the effect of IRAK-M deficiency in airway inflammation under subacute CS exposure, mice were wholly exposed to 7-week CS. There was significantly less infiltration of total inflammatory cells and macrophages in BAL fluids from IRAK-M KO mice compared to WT mice (Figure 5(a)). Compared with IRAK-M KO mice, there was a greater aggregation of leukocytes seen in WT mice (Figure 5(b)). There was a significant decrease in inflammation scores in IRAK-M KO mice compared with WT mice (Figure 5(c)). Consistently, there was a significant inhibition of increased airway resistance induced by subacute CS exposure in IRAK-M KO mice compared to WT mice (Figure 5(d)).

### 3.6. Effect of IRAK-M Ablation on Treg/Th17 in Lungs of Mice under Subacute Exposure to CS

**Figure 6:**
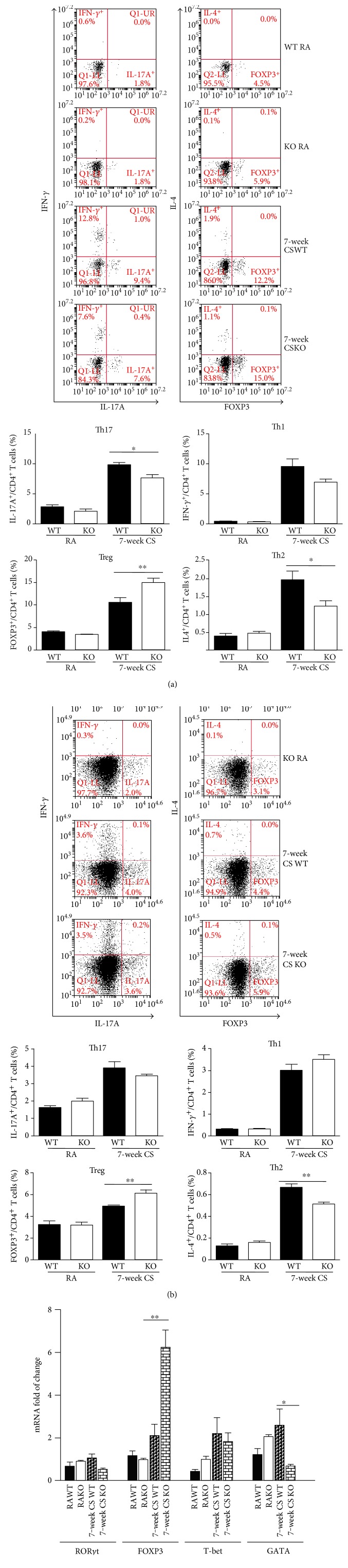
Effect of IRAK-M loss on T subsets after 7-week CS exposure. FACS analysis of Th17 and Treg cells using specific Abs against Th17 and Treg markers in the lung (a) and spleen (b), the percentages of Th17 and Treg were plotted. (c) qRT-PCR analysis of mRNA expression of specific nuclear transcriptional markers for Th1, Th2, Th17, and Treg. Results are expressed as means ± SEM, *n* = 7–8 animals per group, ^∗^*p* < 0.05, ^∗∗^*p* < 0.01, ^∗∗^*p* < 0.001.

FACS analysis showed that there were significantly increased percentages of Tregs in lungs from subacute CS-exposed IRAK-M^−/−^ mice compared with those from similarly treated WT mice; however, there were significantly less percentages of Th17 cells in IRAK-M KO mice than those in WT mice (Figure 6(a)). Compared to WT mice, IRAK-M KO mice showed a significant elevation of Tregs, not Th17, in the spleen after subacute CS exposure (Figure 6(b)). Significantly lower percentages of Th2 cells, not Th1 cells, were seen in mice deficient in IRAK-M compared to that in WT mice after 7-week CS challenge, indicating a role of IKAK-M in inducing more T cell differentiation into Th2 cells, not into Th1 cells, under long-term CS exposure (Figures 6(a) and 6(b)).

Retinoic acid-related orphan receptor *γ*t (ROR*γ*t), a member of the nuclear hormone receptor superfamily, has been identified as a key transcription factor driving the Th17 differentiation program [30]. In contrast, the Treg cells are defined by the expression of the forkhead family transcription factor, FOXP3. qRT-PCR analysis on lung tissues also revealed a significantly higher mRNA expression of FOXP3 in IRAK-M KO mice than that in WT mice following subacute CS exposure (Figure 6(c)). mRNA expression of GATA was significantly induced by subacute CS exposure in WT mice, not in IRAK-M^−/−^ mice. In addition, induction of mRNA expression of RORC was slightly inhibited in IRAK-M KO mice (*p* = 0.09) compared to WT mice (Figure 6(c)).

### 3.7. Effect of IRAK-M Loss on Cytokine Production in Mice Exposed to 7-Week CS

**Figure 7:**
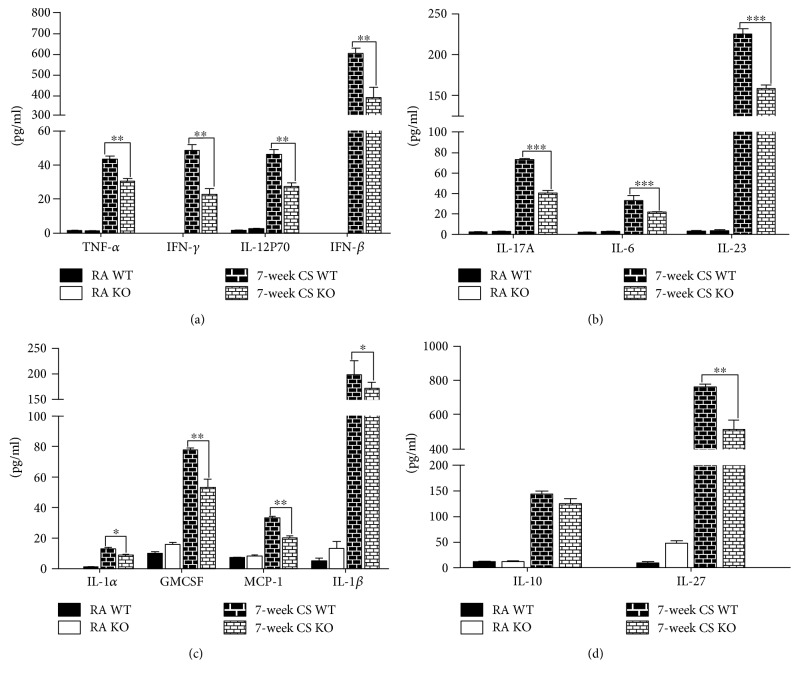
Decreased levels of inflammatory cytokines in lung homogenates in IRAK-M KO mice after 7-week CS exposure. LEGENDplex analysis for levels of cytokines in lung homogenates. (a) Th1-associated cytokines, (b) Th17-related cytokines, (c) proinflammatory cytokines and mediators, (d) anti-inflammatory cytokines. Results are expressed as means ± SEM, *n* = 7 animals per group, ^∗^*p* < 0.05, ^∗∗^*p* < 0.01, ^∗∗∗^*p* < 0.001.

We next determined the concentrations of cytokines in lung homogenates after 7-week CS exposure. As shown in Figure 7, there was significantly lower levels of Th1-associated cytokines (including TNF*α*, IFN*β*, IFN*γ*, and IL-12p70), Th17-related cytokines (including IL-17A, IL-6, and IL-23), and proinflammatory cytokines (including IL-1*α*, IL-1*β*, MCP-1, and GM-CSF) in lung homogenates of IRAK-M KO mice than those in WT mice. There was a significant decrease in the level of anti-inflammatory cytokine IL-27, not IL-10, in lung homogenates from IRAK-M KO mice relative to their WT counterparts in response to subacute CS injury, reflecting the nonspecific inhibition of immune activation under subacute CS stimulation in absence of IRAK-M.

### 3.8. Effect of IRAK-M Deficiency on Surface Expression of Costimulatory Molecules Induced by Subacute CS Exposure in Mice

**Figure 8:**
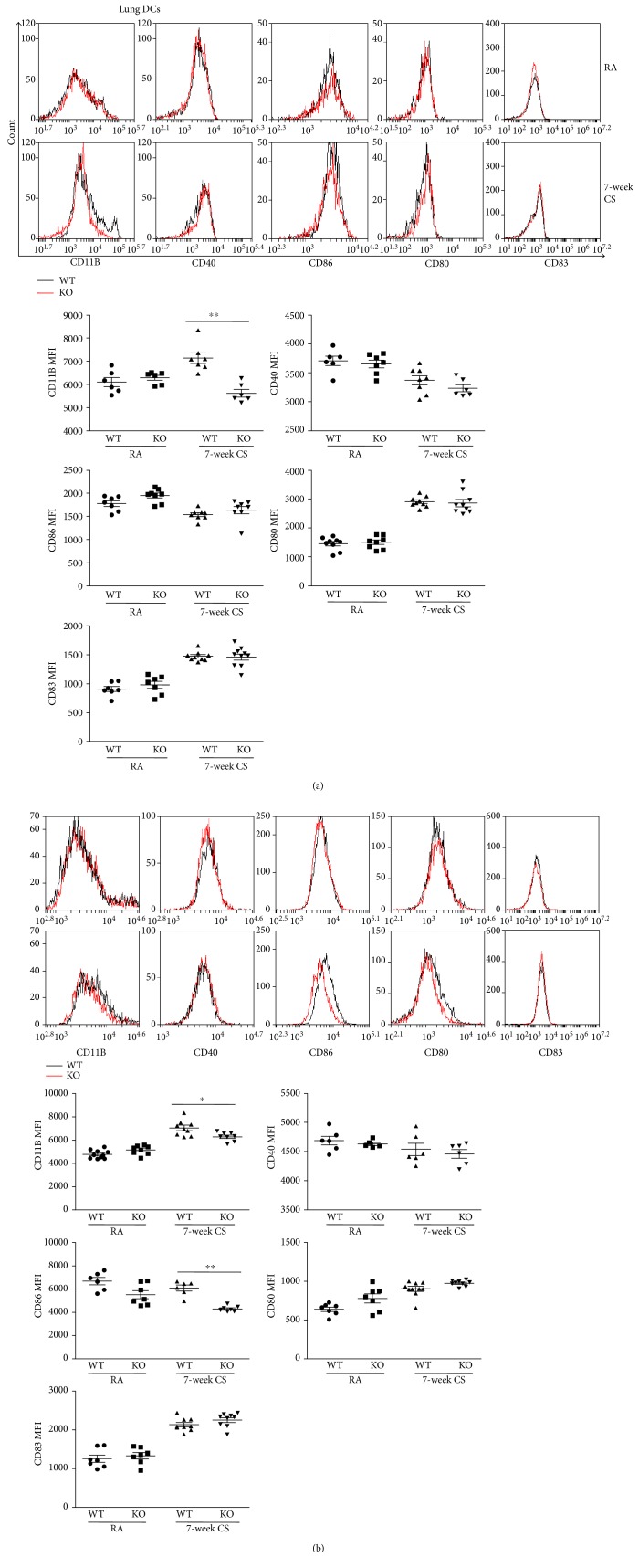
Effect of IRAK-M loss on surface expression of costimulatrory molecules on lung DCs and macrophages after 7-week CS exposure. FACS analysis of surface expression of costimulatory molecules on (a) lung DCs and (b) macrophages using specific Abs against the indicated costimulatory molecule. Mean fluorescence intensity of indicated costimulatory molecules was plotted. Results are expressed as means ± SEM, ^∗^*p* < 0.05, ^∗∗^*p* < 0.01.

Compared to air-exposed mice, expression of costimulatory molecule CD11b was lower on lung DCs from both IRAK-M KO and WT mice after 7-week CS exposure. There were no significant differences in expression of other costimulatory molecules by DCs between two types of mice (Figure 8(a)).

Chronic exposure to CS constitutively activates alveolar macrophages [31]. However, our FACS analysis showed that expression of CD11b and CD86 by lung macrophages was significantly lower in IRAK-M KO mice than that in WT mice after 7-week CS exposure (Figure 8(b)).

## 4. Discussion

IRAK-M is a MyD88-dependent inhibitor of TLR signaling and reported to display different functions depending on the milieu where it is expressed [6, 32]. We firstly to our knowledge examined the effects of IRAK-M on airway inflammation induced by acute CS exposure (3 days) followed by LPS inhalation and subacute CS exposure (7 weeks) using IRAK-M KO mice and WT littermates. In this present investigation, we showed IRAK-M expression temporally and spatially affected the pathological outcomes in the CS-induced airway inflammation with or without LPS. Compared with WT counterparts, mice deficient in IRAK-M showed significantly aggravated airway inflammation and more number of Th17 cells and less number of Treg in lungs after acute CS exposure followed by LPS challenge. Conversely, subacutely CS-exposed IRAK-M^−/−^ mice manifested significantly attenuated infiltration of inflammatory cells into the airways and less number of Th17 cells and more Tregs in lungs compared to similarly administrated WT mice.

Exposure to CS is a risk factor for lung infection, and bacterial infections substantially contribute to exacerbations of COPD [33]. The airway inflammatory response in lung pathology induced by CS or bacteria is associated with infiltration of leukocytes, particularly, neutrophils and lymphocytes [34, 35]. We investigated the effect of IRAK-M loss on host defense against LPS in mice exposed to 3-day CS. IRAK-M KO mice displayed more severe lung inflammation and significantly increased accumulation of neutrophils and lymphocytes in the airways compared with WT mice. Consistently, concentrations of Th1-associated cytokines (IFN*β*, IFN*γ*, and IL-12p70), proinflammatory cytokine TNF*α*, and Th17-related cytokines (IL-17A and IL-6) in BAL fluids were significantly higher in IRAK-M KO mice than those in the WT mice. Because IRAK-M expression is found in airway epithelium and macrophages [7, 8], we cautiously think that significant infiltration of neutrophils and lymphocytes was a downstream effect after administrated with LPS and acute CS exposure possibly mediated by IRAK-M.

Th17/Treg imbalance has been implicated in the immunopathology of COPD [1]. Th17 cells, a distinct lineage of activated CD4^+^ T cells, modulate tissue inflammation by producing IL-17A and IL-17F [36]. They also mediate immunity against extracellular pathogens [1]. Regulatory T cells (Treg) are subsets of CD4^+^ T cells with immunoregulatory functions, which suppress inflammation by producing anti-inflammatory cytokines such as IL-10 [37].

Smokers with COPD had significantly fewer Treg cells in the lungs, less mRNA for FOXP3, and less IL-10 secretion from the whole lung than controls [38]. Consistent with the previous reports, we found an increase of the proportion of Th17 cells and a decrease of Tregs in both lungs and spleens from IRAK-M KO mice than WT mice after CS exposure plus LPS challenge.

Interestingly, our data showed that IRAK-M promoted airway inflammation induced by subacute exposure to CS in a mouse model. This is evident from the significant alleviation in infiltration of inflammatory cells around the airways and in pulmonary parenchyma, airway inflammatory response (for total BAL inflammatory cells and macrophages), and airway resistance seen in IRAK-M KO mice compared to those in WT mice. IRAK-M KO mice exposed to subacute CS showed decreased levels of Th17-related cytokines (including IL-6, IL-17A, and IL-23) and Th1-associated cytokines (e.g., TNF*α*, IFN*β*, IFN*γ*, and IL-12p70), which may associate with reduced airway inflammation in these knockout mice. Consistent with the previous report demonstrating expression of Th1-type proinflammatory cytokines regulated by IRAK-M [39], our current data also showed that IRAK-M deficiency resulted in lower levels of Th1-type cytokines (TNF*α*, IFN*β*, IFN*γ*, IL-12p70, and IL-12) and Th17-related cytokines (IL-17A, IL-6, and IL-23) in BAL fluid after 7-week CS exposure. Airway epithelial cells can be abnormally activated by CS and play an important role in the pathology of airway injury [40]. We also observed decreased expression of airway epithelium-related proinflammatory cytokines and chemokines secreted (including IL-1*α*, IL-1*β*, MCP-1, and GM-CSF) in the lungs of IRAK-M KO mice after subacute CS exposure, suggesting the inhibition of overactivation of airway epithelial cells in absence of IRAK-M. Taken together, IRAK-M deficiency alleviated lung inflammation possibly through regulating T cell subpopulations infiltrated in the lungs after subacute CS challenge.

By presenting antigen/MHC and costimulatory molecules to T cells, airway DCs play an important role in inducing activation and differentiation of naïve and effector CD4^+^ and CD8^+^ T cells, which both of these two cell types are involved in COPD pathogenesis [28]. Furthermore, disruptive airway epithelial barrier caused by CS makes lung DCs exposed to environmental challenges.

CS was reported to promote accumulation and survival of matured DCs in lung tissues from COPD patients [41]. Significant increases in costimulatory molecule expression by mature DCs contribute to an inappropriate immune response in the pathogenesis of COPD [42]. Pulmonary DCs of smoke-exposed mice were shown to be activated with surface expression of MHC II and costimulatory molecules CD40 and CD86 being significantly upregulated under 24-week CS exposure [43]. Quite interestingly, recent evidence has shown that long-term cigarette smoking extract (CSE) exposure downregulated CD11c/MHCII, CD83, CD86, and CD40 expression by DCs; however, short-term CSE stimulation upregulated surface expression of MHCII, CD83, CD86, and CD40 by lung DCs [44]. IRAK-M expression has been reported to inhibit DC activation and production of proinflammatory cytokines in response to *Helicobacter pylori* [45]. Airway macrophages function as the innate defense of the airways and drive the pulmonary immune response to CS and infection; thus, airway macrophages play important role in the pathogenesis of CS-induced airway inflammatory diseases [35]. In addition to its effects on DCs, IRAK-M modulates phenotype and function of mononuclear cells [17]. We used FACS analysis to reflect the effect of IRAK-M on expression of costimulatory molecules by DCs and macrophages under acute/subacute CS exposure. Under the acute CS exposure followed by LPS challenge, IRAK-M KO mice showed significant upregulation of costimulatory molecules CD40 and CD86 in lung DCs or macrophages compared with similarly treated WT mice. However, after 7-week CS exposure, significantly higher expression of CD11b by DCs and lower expression of CD86 by lung macrophages were seen in IRAK-M KO mice than those in WT mice. These observations might be attributed to the regulatory effect of IRAK-M on DCs and macrophages in the lungs, depending on the duration of stimulation and type of stimulus.

## 5. Conclusions

In the present study, we demonstrated that the role of IRAK-M signaling could differ between LPS-induced lung injury after short CS exposure and subchronic CS-mediated lung damage in a mouse model. IRAK-M provided airway protection against combined exposure to LPS and acute CS by influencing Treg/Th17 imbalance skewing to Th17 and upregulating expression of costimulatory molecules CD40 and CD86 by DCs and macrophages. In contrast, IRAK-M played a proinflammatory role in airway pathology under 7-week CS exposure by influencing Treg/Th17 imbalance skewing to Treg and downregulating expression of costimulatory molecules CD11b and CD86 by macrophages. An appropriate modulation of IRAK-M in airway inflammation induced by the duration of CS insult or type of stimulus might be helpful in searching for new targets to CS-associated airway pathology.

## Figures and Tables

**Table 1:** Sequences for real-time quantitative RT-PCR primers.

qRT-PCR genes	Accession number	Primer sequences (5′—3′)
T-bet	NM_019507.2	F: GTTCAACCAGCACCAGACAGAG
		R: TGGTCCACCAAGACCACATC
GATA3	NM_008091.3	F: GGATGTAAGTCGAGGCCCAAG
		R: ATTGCAAAGGTAGTGCCCGGTA
RORC	NM_001293734.1	F: GCTCCATATTTGACTTTTCCCACT
		R: GATGTTCCACTCTCCTCTTCTCTTG
FOXP3	NM_001199348.1	F: AGTGCCTGTGTCCTCAATGGTC
		R: AGGGCCAGCATAGGTGCAAG
GAPDH	NM_001289726.1	F:TTGTCTCCTGCGACTTCAACA
		R:TGGTCCAGGGTTTCTTACTCC

## References

[1] Brusselle G. G., Joos G. F., Bracke K. R. (2011). New insights into the immunology of chronic obstructive pulmonary disease. *Lancet*.

[2] Kang M. J., Lee C. G., Lee J. Y. (2008). Cigarette smoke selectively enhances viral PAMP- and virus-induced pulmonary innate immune and remodeling responses in mice. *The Journal of Clinical Investigation*.

[3] Smyth L. J., Starkey C., Gordon F. S., Vestbo J., Singh D. (2008). Cd8 chemokine receptors in chronic obstructive pulmonary disease. *Clinical and Experimental Immunology*.

[4] Smyth L. J., Starkey C., Vestbo J., Singh D. (2007). Cd4-regulatory cells in COPD patients. *Chest*.

[5] Wang H., Peng W., Weng Y. (2012). Imbalance of Th17/Treg cells in mice with chronic cigarette smoke exposure. *International Immunopharmacology*.

[6] Hubbard L. L., Moore B. B. (2010). IRAK-M regulation and function in host defense and immune homeostasis. *Infectious Disease Reports*.

[7] Kobayashi K., Hernandez L. D., Galan J. E., Janeway C. A., Medzhitov R., Flavell R. A. (2002). IRAK-M is a negative regulator of Toll-like receptor signaling. *Cell*.

[8] Balaci L., Spada M. C., Olla N. (2007). IRAK-M is involved in the pathogenesis of early-onset persistent asthma. *American Journal of Human Genetics*.

[9] Besnard A. G., Sabat R., Dumoutier L. (2011). Dual role of IL-22 in allergic airway inflammation and its cross-talk with IL-17a. *American Journal of Respiratory and Critical Care Medicine*.

[10] Sonnenberg G. F., Nair M. G., Kirn T. J., Zaph C., Fouser L. A., Artis D. (2010). Pathological versus protective functions of IL-22 in airway inflammation are regulated by IL-17a. *The Journal of Experimental Medicine*.

[11] Kobayashi T., Tanaka K., Fujita T. (2015). Bidirectional role of IL-6 signal in pathogenesis of lung fibrosis. *Respiratory Research*.

[12] Chen W., Saxena A., Li N. (2012). Endogenous IRAK-M attenuates postinfarction remodeling through effects on macrophages and fibroblasts. *Arteriosclerosis, Thrombosis, and Vascular Biology*.

[13] Wu Q., Jiang D., Smith S. (2012). IL-13 dampens human airway epithelial innate immunity through induction of IL-1 receptor-associated kinase M. *The Journal of Allergy and Clinical Immunology*.

[14] Wu Q., van Dyk L. F., Jiang D. (2013). Interleukin-1 receptor-associated kinase M (IRAK-M) promotes human rhinovirus infection in lung epithelial cells via the autophagic pathway. *Virology*.

[15] Deng J. C., Cheng G., Newstead M. W. (2006). Sepsis-induced suppression of lung innate immunity is mediated by IRAK-M. *The Journal of Clinical Investigation*.

[16] Seki M., Kohno S., Newstead M. W. (2010). Critical role of IL-1 receptor-associated kinase-M in regulating chemokine-dependent deleterious inflammation in murine influenza pneumonia. *Journal of Immunology*.

[17] Ballinger M. N., Newstead M. W., Zeng X. (2015). IRAK-M promotes alternative macrophage activation and fibroproliferation in bleomycin-induced lung injury. *Journal of Immunology*.

[18] Nie L., Xiang R., Zhou W., Lu B., Cheng D., Gao J. (2008). Attenuation of acute lung inflammation induced by cigarette smoke in CXCR3 knockout mice. *Respiratory Research*.

[19] Ghorani V., Boskabady M. H., Khazdair M. R., Kianmeher M. (2017). Experimental animal models for COPD: a methodological review. *Tobacco Induced Diseases*.

[20] Le Quement C., Guenon I., Gillon J. Y. (2008). The selective MMP-12 inhibitor, AS111793 reduces airway inflammation in mice exposed to cigarette smoke. *British Journal of Pharmacology*.

[21] Leclerc O., Lagente V., Planquois J. M. (2006). Involvement of MMP-12 and phosphodiesterase type 4 in cigarette smoke-induced inflammation in mice. *The European Respiratory Journal*.

[22] Wang L., Xu Z., Chen B. (2017). The role of vascular endothelial growth factor in small-airway remodelling in a rat model of chronic obstructive pulmonary disease. *Scientific Reports*.

[23] Lin Y., Yan H., Xiao Y. (2011). Attenuation of antigen-induced airway hyperresponsiveness and inflammation in CXCR3 knockout mice. *Respiratory Research*.

[24] Suzaki Y., Hamada K., Nomi T. (2008). A small-molecule compound targeting CCR5 and CXCR3 prevents airway hyperresponsiveness and inflammation. *The European Respiratory Journal*.

[25] Willis C. R., Siegel L., Leith A. (2015). Il-17ra signaling in airway inflammation and bronchial hyperreactivity in allergic asthma. *American Journal of Respiratory Cell and Molecular Biology*.

[26] Kearley J., Silver J. S., Sanden C. (2015). Cigarette smoke silences innate lymphoid cell function and facilitates an exacerbated type I interleukin-33-dependent response to infection. *Immunity*.

[27] Turnis M. E., Song X. T., Bear A. (2010). IRAK-M removal counteracts dendritic cell vaccine deficits in migration and longevity. *Journal of Immunology*.

[28] Freeman C. M., Curtis J. L. (2017). Lung dendritic cells: shaping immune responses throughout chronic obstructive pulmonary disease progression. *American Journal of Respiratory Cell and Molecular Biology*.

[29] Nolan A., Kobayashi H., Naveed B. (2009). Differential role for CD80 and CD86 in the regulation of the innate immune response in murine polymicrobial sepsis. *PLoS One*.

[30] Ivanov I. I., McKenzie B. S., Zhou L. (2006). The orphan nuclear receptor RORgammat directs the differentiation program of proinflammatory IL-17+ T helper cells. *Cell*.

[31] Kaku Y., Imaoka H., Morimatsu Y. (2014). Overexpression of CD163, CD204 and CD206 on alveolar macrophages in the lungs of patients with severe chronic obstructive pulmonary disease. *PLoS One*.

[32] Du J., Nicolaes G. A., Kruijswijk D., Versloot M., van der Poll T., van 't Veer C. (2014). The structure function of the death domain of human IRAK-M. *Cell Communication and Signaling: CCS*.

[33] Sethi S., Murphy T. F. (2008). Infection in the pathogenesis and course of chronic obstructive pulmonary disease. *The New England Journal of Medicine*.

[34] Thatcher T. H., McHugh N. A., Egan R. W. (2005). Role of CXCR2 in cigarette smoke-induced lung inflammation. *American Journal of Physiology Lung Cellular and Molecular Physiology*.

[35] Gadgil A., Duncan S. R. (2008). Role of T-lymphocytes and pro-inflammatory mediators in the pathogenesis of chronic obstructive pulmonary disease. *International Journal of Chronic Obstructive Pulmonary Disease*.

[36] Miossec P., Korn T., Kuchroo V. K. (2009). Interleukin-17 and type 17 helper T cells. *The New England Journal of Medicine*.

[37] Pandiyan P., Zhu J. (2015). Origin and functions of pro-inflammatory cytokine producing Foxp3+ regulatory T cells. *Cytokine*.

[38] Lee S. H., Goswami S., Grudo A. (2007). Antielastin autoimmunity in tobacco smoking-induced emphysema. *Nature Medicine*.

[39] Jeyanathan M., McCormick S., Lai R. (2014). Pulmonary M. tuberculosis infection delays Th1 immunity via immunoadaptor DAP12-regulated IRAK-M and IL-10 expression in antigen-presenting cells. *Mucosal Immunology*.

[40] Thorley A. J., Tetley T. D. (2007). Pulmonary epithelium, cigarette smoke, and chronic obstructive pulmonary disease. *International Journal of Chronic Obstructive Pulmonary Disease*.

[41] Vassallo R., Walters P. R., Lamont J., Kottom T. J., Yi E. S., Limper A. H. (2010). Cigarette smoke promotes dendritic cell accumulation in COPD; a lung tissue research consortium study. *Respiratory Research*.

[42] Freeman C. M., Martinez F. J., Han M. K. (2009). Lung dendritic cell expression of maturation molecules increases with worsening chronic obstructive pulmonary disease. *American Journal of Respiratory and Critical Care Medicine*.

[43] D'Hulst A. I., Vermaelen K. Y., Brusselle G. G., Joos G. F., Pauwels R. A. (2005). Time course of cigarette smoke-induced pulmonary inflammation in mice. *The European Respiratory Journal*.

[44] Givi M. E., Folkerts G., Wagenaar G. T., Redegeld F. A., Mortaz E. (2015). Cigarette smoke differentially modulates dendritic cell maturation and function in time. *Respiratory Research*.

[45] Shiu J., Czinn S. J., Kobayashi K. S., Sun Y., Blanchard T. G. (2013). IRAK-M expression limits dendritic cell activation and proinflammatory cytokine production in response to Helicobacter pylori. *PLoS One*.

